# Telecoaching plus a portion control plate for weight care management: a randomized trial

**DOI:** 10.1186/s13063-015-0880-1

**Published:** 2015-07-30

**Authors:** Jill M. Huber, Joshua S. Shapiro, Mark L. Wieland, Ivana T. Croghan, Kristen S. Vickers Douglas, Darrell R. Schroeder, Julie C. Hathaway, Jon O. Ebbert

**Affiliations:** Division of Primary Care Internal Medicine, Department of Medicine, Rochester, MN 55905 USA; Division of Internal Medicine, Department of Medicine, Rochester, MN 55905 USA; Department of Psychiatry and Psychology, Mayo Clinic, Rochester, MN 55905 USA; Division of Biomedical Statistics and Informatics, Department of Health Sciences Research, Mayo Clinic, Rochester, MN 55905 USA; Patient Education and Consulting Services, Mayo Clinic, Rochester, MN 55905 USA; Mayo Clinic, 200 First Street Southwest, Rochester, MN 55905 USA

**Keywords:** Obesity, Telecoaching, Portion control plate, Primary care, Patient-centered medical home

## Abstract

**Background:**

Obesity is a leading preventable cause of death and disability and is associated with a lower health-related quality of life. We evaluated the impact of telecoaching conducted by a counselor trained in motivational interviewing paired with a portion control plate for obese patients in a primary care setting.

**Methods:**

We conducted a randomized, clinical trial among patients in a primary care practice in the midwestern United States. Patients were randomized to either usual care or an intervention including telecoaching with a portion control plate. The intervention was provided during a 3-month period with follow-up of all patients through 6 months after randomization. The primary outcomes were weight, body mass index (BMI),waist circumference, and waist to hip ratio measured at baseline, 6, 12, 18, and 24 weeks. Secondary outcomes included measures assessing eating behaviors, self-efficacy, and physical activity at baseline and at 12 and 24 weeks.

**Results:**

A total of 1,101 subjects were pre-screened, and 90 were randomly assigned to telecoaching plus portion control plate (n = 45) or usual care (n = 45). Using last-value carried forward without adjustment for baseline demographics, significant reductions in BMI (estimated treatment effect -0.4 kg/m^2^, *P* = .038) and waist to hip ratio (estimated treatment effect -.02, *P* = .037) at 3 months were observed in the telecoaching plus portion control plate group compared to usual care. These differences were not statistically significant at 6 months. In females, the telecoaching plus portion control plate intervention was associated with significant reductions in weight and BMI at both 3 months (estimated treatment effect -1.6 kg, *P* = .016 and -0.6 kg/m^2^, *P* = .020) and 6 months (estimated treatment effect -2.3 kg, *P* = .013 and -0.8 kg/m^2^, *P* = .025). In males, the telecoaching plus portion control intervention was associated with a significant reduction in waist to hip ratio at 3 months (estimated treatment effect -0.04, *P* = .017), but failed to show a significant difference in weight and BMI.

**Conclusion:**

Telecoaching with a portion control plate can produce positive change in body habitus among obese primary care patients; however, changes depend upon sex.

**Trial registration:**

ClinicalTrials.gov NCT02373878, 13 February 2015. https://clinicaltrials.gov/ct2/show/NCT02373878.

**Electronic supplementary material:**

The online version of this article (doi:10.1186/s13063-015-0880-1) contains supplementary material, which is available to authorized users.

## Background

Obesity is a leading cause of preventable death and disability in the United States [1], a lower health-related quality of life [2], and contributes to chronic disease burdens of hypertension, hyperlipidemia, osteoarthritis, cancer, diabetes mellitus, and sleep apnea. Medical costs for people who are obese are $1429 higher per year than those of normal weight and total $147 billion annually [3].

The American Medical Association, American College of Physicians, and the United States Preventive Services Task Forces have published guidelines that define obesity as a disease and recommend intensive physician-patient behavioral modification for obese patients [1, 2, 4–7]. However, lack of physician disease recognition, time, and skills make implementation of these recommendations unlikely [8–10]. Furthermore, existing trials of obesity counseling by primary care physicians have demonstrated mixed results [11–13]. A large meta-analysis of primary care diet and physical activity interventions recently showed that the most effective interventions utilize a combined lifestyle approach and require intensive patient contact spread over several months [14]. This intensive patient contact may be best achieved by utilizing allied healthcare providers as has been demonstrated with successful clinical weight loss interventions administered by a registered dietician [15, 16], a medical assistant [17], or by a counselor using motivational interview techniques [18].

Recently, there has also been an increased interest in maintaining patient contact via information technology such as email, telephone, and online messaging [19, 20]. Telephone counseling has been associated with positive behavior changes such as increasing physical activity and improving nutrition [21] while also being associated with significant weight loss [16]. Additionally, group telephone counseling via conference calls conducted by counselors has also demonstrated promising results [22]. Although telecoaching has proven efficacious, it remains poorly understood how it lends itself to a combined lifestyle approach in weight loss interventions.

We conducted a pilot study of a weight loss intervention utilizing telecoaching conducted by a counselor trained in motivational interviewing for obese patients in a primary care setting. Additionally, patients received a portion control plate, which previously had been shown to be helpful for monitoring food portions [23]. We hypothesized that an intervention utilizing a combined lifestyle approach to weight management would be feasible and result in significant weight loss.

## Methods

### Study overview

We conducted a randomized clinical trial evaluating the effectiveness of telecoaching combined with a portion control plate. Telecoaching was provided during a 3-month intervention period with follow-up through 6 months after randomization. The primary study outcomes were weight reduction, BMI, waist circumference, and waist to hip ratio, measured at 6, 12, 18, and 24 weeks. Secondary outcomes included measures assessing eating behaviors, self-efficacy, and physical activity collected at 12 and 24 weeks. Enrollment took place between May 2011 and June 2012. This trial was performed in accordance with the CONSORT guidelines for randomized controlled trials (see Additional file 1 and 2 for details).

### Setting

The study was conducted at a large academic primary care practice in the Midwestern United States. The practice provides primary care to approximately 130,000 patients.

### Study participants

Participants were eligible for the study if they were between the ages of 18 and 55 years with a body mass index ≥30 and ≤39.9 kg/m^2^ (obesity class I and II) and were motivated to pursue weight loss. We selected the upper age limit of 55 years because we were most interested in determining if the intervention was effective in a population at risk for complications related to overweight and obesity who may not yet be suffering these consequences. Participants were excluded if they had a significant health condition (for example, recent myocardial infarction, untreated hypertension, bipolar disorder, *etcetera*), had undergone bariatric surgery, were pregnant, or were utilizing an investigational weight loss medication. This study was approved by the Institutional Review Board of Mayo Clinic prior to patient contact: reference number 11-001395.

### Study enrollment

Study personnel performed a limited chart review based on BMI and age eligibility criteria to identify primary care patients eligible for the study. For potentially eligible participants who had an upcoming appointment, their primary care providers were alerted of their eligibility. The study was then introduced at the clinic visit with referral of those interested to study personnel for consideration of enrollment. For eligible participants who did not have an upcoming appointment in primary care, a recruitment letter was sent with instructions on how to contact study personnel for enrollment. All interested eligible patients underwent initial pre-screening via telephone by study personnel. Potential subjects then met with study personnel to review the study protocol in detail. Informed consent was obtained from all participants (or next of kin) prior to enrolment in the trial.

### Randomization

A computer generated randomization schedule was created using blocks of size 4 to ensure that treatment assignment was balanced across groups over the course of the enrollment period. Using this randomization schedule, individuals who did not have any subject contact for the present study prepared randomization envelopes, which were labeled according to subject ID number and contained an index card indicating the treatment assignment for the given subject. At the time of enrollment, a subject was assigned the next sequential subject ID number, and the appropriate sealed envelope was opened to reveal the subject’s randomized treatment assignment. The authors were blinded to randomization. The interventionist was not blinded to study assignment given the nature of the intervention Additional files 3.

### Intervention

In the intervention group, participants received a portion control plate with instructions on use along with telecoaching. Telecoaching involved telephone counseling on lifestyle modification from a single master’s level female counselor trained in motivational interviewing and wellness coaching.

The wellness coach proactively contacted the participants every 2 weeks for 3 months for a total of seven phone calls. Motivational interviewing was the framework for intervention delivery. Motivational interviewing is a directive, patient-centered counseling style for eliciting behavior change by helping patients to explore and resolve ambivalence [24], with efficacy data for weight loss in face-to-face clinical settings [25]. The focus of the discussion was an improvement in lifestyle by identifying barriers to incorporating healthy behaviors and problem solving to overcome these, collaborative goal setting, and progress toward patient-identified behavior change goals. Specific strategies were utilized to focus on improving diet and physical activity. These included, giving information about the “500 calorie challenge,” which is a strategy for reducing daily caloric intake by 500 calories*.* Additionally, recommendations were given for incorporating routine self-weighing, tools for monitoring and documenting intake, and regular physical activity with a goal of 150 min of moderate intensity exercise per week.

Average phone call duration was approximately 20 minutes with the first and final calls being slightly longer. The format of the coaching session was a “check in,” focusing on progress towards behavior change goals from the previous call, discussion around successes and barriers, problem solving, and collaboratively setting an action plan for the upcoming 2 weeks. The usual care group received institutional pamphlets on healthy eating and exercise habits.

### Study measures

Participants in both groups were evaluated in the clinic at baseline and at 6, 12, 18, and 24 weeks to obtain weight, BMI, waist circumference, and waist to hip ratio. Participants also completed measures assessing eating behaviors and physical activity at baseline and at 12 and 24 weeks.

The primary outcomes included body weight, BMI, waist circumference, and waist to hip ratio. Body weight was measured by a digital scale calibrated on a regular basis using certified weights. Participants were measured with their shoes and heavy outer garments removed and pockets emptied. The BMI was calculated from measured height and weight (kg/height [meters]^2^). Waist circumference measurement was standardized utilizing a measuring tape held in the horizontal plane around the abdomen at the iliac crest and taken at the end of a normal expiration with the tape snug and not compressing the skin. Waist to hip ratio was calculated by dividing the waist circumference measurement by the measurement obtained at the narrowest part of the hips.

Secondary outcomes included physical activity level, dietary changes, self-efficacy, social support, and constructs of behavioral change. Physical activity was measured via two separate surveys. The Seven-Day Physical Activity Recall [26] is a five-item assessment of physical activity over the previous 7 days that characterizes sleep, light, moderately hard, hard, and very hard activity. Results are reported as total daily energy expenditure (kcal/day). The International Physical Activity Questionnaire (IPAQ) [27] is a seven-question self-report measure of physical activity that has been shown valid and repeatable in very diverse settings throughout 12 different countries. The questionnaire assesses frequency and duration of walking, moderate-intensity activity, and vigorous-intensity activity over a 1-week period, with results reported in total Metabolic Equivalents (METs) per week.

Dietary changes were measured via the Food Frequency Questionnaire (FFQ), which is a self-administered food questionnaire that asks the participant to report frequency of consumption and portion size of 125 common food items over a given time [28, 29]. This was administered electronically.

The Eating Inventory [30] measured dietary restraint, disinhibition, and hunger. Research has demonstrated that scores can improve following obesity treatment, and that scores predict outcome to obesity treatment [31]. Additionally, we utilized the Weight Efficacy Life-Style Questionnaire (WEL) [32], which is a 20-item eating self-efficacy scale consisting of a total score and five situational factors: negative emotions, availability, social pressure, physical discomfort, and positive activities. Participants rated their confidence in being able to successfully resist the urge to eat using a 10-point scale ranging from 0 (not confident) to 9 (very confident) [33]. Improvements in eating self-efficacy have been associated with both greater weight loss as well as improved performance in weight control behaviors during intensive treatments [34]. We measured social support with the Weight Management Support Inventory (WMSI) [35], which assesses four dimensions of social support (emotional, instrumental, informational, and appraisal). The WMSI allows measurement of baseline levels of support for weight management and verifies changes in support via intervention. Finally, we measured commonly accepted constructs of behavioral change with the Neis Behavior Change Scale (NEIS), which is a 16-item questionnaire that evaluates three theoretical constructs: (a) goal setting, (b) restructuring plans, and (c) relapse prevention and maintenance. These constructs have previously been identified as important aspects of interventions, and this scale has been shown to have both internal and test-retest reliability in assessing these [36].

### Sample size and statistical analysis

This randomized trial was a pilot study, and a power analysis to determine number of subjects needed for statistical significance was not performed. Data are presented as mean ± SD or median (25th, 75th) for continuous variables and frequency percentages for nominal variables. Body size measurements at 3 and 6 months were compared between groups using analysis of covariance. For these models, the follow-up measurement was the dependent variable, treatment group was the independent variable and the baseline value of the measurement was included as the covariate. These analyses were performed using only subjects with complete data, and using the approach of last value carried forward. For these analyses, the results are summarized by presenting the estimated treatment effect and corresponding 95 % confidence interval. From initial comparisons of baseline characteristics, the distribution of males and females was found to differ significantly between treatment groups. For this reason, post-hoc analyses were performed which included sex as a covariate, and supplemental analyses were performed separately for males and females. Due to skewed distributions, the change from baseline to 3 months for secondary outcomes was compared between groups using the rank sum test. In all cases, two-sided tests were performed with *P* values ≤ .05 considered statistically significant.

## Results

### Enrollment and follow-Up

Of the 1101 subjects screened, 106 passed a telephone pre-screen. Of these 106, 92 attended a consent/screen visit, and 90 were randomly assigned to telecoaching plus portion control plate (n = 45) or usual care (n = 45) (Table 1). Although the majority of both treatment groups were female, the percentage of males in the telecoaching plus portion control group was significantly lower than that for the usual care group (16 % versus 36 %; *P* = .030). Other baseline characteristics were similar between groups. Fourteen subjects discontinued the study, as they were not present for the 6-month follow-up visit resulting in an overall study completion rate of 84 % (82 % for telecoaching plus portion control, 87 % for usual care).

**Table 1:** Baseline demographic characteristics

	Telecoaching + portion control	Usual care
	(N = 45)	(N = 45)
Age, years		
mean ± SD	48.3 ± 12.3	47.4 ± 14.1
range	20 to 65	18 to 70
Sex, no. (%)		
Male	7 (16)	16 (36)
Female	38 (84)	29 (64)
Race/ethnicity, no. (%)		
White, non-Hispanic	43 (96)	41 (91)
Other	2 (4)	4 (9)
Marital status, no. (%)		
Married/living as married	26 (58)	34 (76)
Separated/divorced	2 (4)	4 (9)
Never married	13 (29)	7 (16)
Widowed	4 (9)	0 (0)
Number of people in household, no. (%)		
1	11 (24)	7 (16)
2	13 (29)	21 (47)
3	7 (16)	6 (13)
4 or more	14 (31)	11 (24)
Education, no. (%)		
Less than high school	1 (2)	0 (0)
High school graduate	8 (18)	3 (7)
Some college	17 (38)	17 (38)
4-year college degreeor more	19 (42)	25 (56)
Work status, no. (%)		
Full time	34 (76)	29 (66)
Part time	6 (13)	7 (16)
Unemployed	2 (4)	0 (0)
Retired	1 (2)	8 (18)
Current tobacco use, no. (%)	4 (9)	1 (2)
Diabetes, no. (%)	3 (7)	4 (9)
Prior treatment fordepression, no. (%)	14 (31)	13 (29)
Prior treatment for alcoholism, no. (%)	2 (4)	2 (4)
Weight,, mean ± SD, kg	99.6 ± 14.0	103.6 ± 18.9
BMI, mean ± SD	36.5 ± 4.2	36.1 ± 3.9
Waist, mean ± SD, cm	108.6 ± 9.3	112.4 ± 13.3
Waist-hip ratio, mean ± SD	0.88 ± 0.10	0.91 ± 0.11
FFQ, total calories,		
median (25th, 75th)	2271 (1531, 3054)	2366 (1865, 3725)
IPAQ, total METs,		
median (25th, 75th)	1760 (998, 4666)	1440 (680, 4596)
WEL, total score,		
median (25th, 75th)	129 (110, 139)	116 (95, 136.5)
Nies Behavior Change, median (25th, 75th)		
Goal setting	20.0 (17.5, 23.0)	20.0 (17.5, 24.0)
Restructuring plans	14.0 (13.0, 15.0)	14.0 (13.0, 16.0)
Relapse prevention	18.0 (16.0, 21.0)	18.5 (15.0, 21.0)

*BMI* body mass index (calculated as weight in kilograms divided by height in meters squared), *FFQ* Food Frequency Questionnaire, *IPAQ* International Physical Activity Questionnaire, *WEL* Weight Efficacy Life-Style Questionnaire

### Body measurement outcomes

In the primary analysis using last-value carried forward without adjustment for baseline demographics, significant reductions in BMI (estimated treatment effect -0.4 kg/m^2^, *P* = .038) and waist to hip ratio (estimated treatment effect -.02, *P* = .037) at 3 months were observed in the telecoaching plus portion control plate group compared to usual care (Table 2). These differences were not statistically significant at 6 months.

**Table 2:** Body size change from baseline to 3 and 6 months, overall and according to sex

	Change from baseline									
	Telecoaching + portion control		Usual care		Complete case estimated treatment effect			Last value carried forward estimated treatment effect		
	N	Mean ± SD	N	Mean ± SD	Est	(95 % C.I.)	P*	Est	(95 % C.I.)	*P* value*
All Subjects										
3-months										
Weight, kg	38	−2.2 ± 3.1	40	−1.0 ± 2.0	−1.2	(-2.4, -0.1)	0.045	−0.9	(-1.9, +0.1)	0.091
BMI, kg/m^2^	38	−0.9 ± 1.2	40	−0.3 ± 0.7	−0.5	(-1.0, -0.1)	0.020	−0.4	(-0.8, -0.0)	0.038
Waist, cm	38	−3.2 ± 3.6	39	−1.9 ± 2.6	−1.3	(-2.7, +0.1)	0.072	−0.9	(-2.2, +0.4)	0.180
Waist-hip ratio	38	−0.02 ± 0.05	39	−0.00 ± 0.03	−0.02	(-0.04, -0.00)	0.037	−0.02	(-0.04, -0.00)	0.037
6-months										
Weight, kg	37	−2.6 ± 4.4	39	−1.1 ± 3.7	−1.5	(-3.3, +0.4)	0.118	−1.0	(-2.6, +0.6)	0.225
BMI, kg/m^2^	37	−1.0 ± 1.7	39	−0.4 ± 1.3	−0.7	(-1.4, -0.1)	0.038	−0.5	(-1.1, +0.1)	0.093
Waist, cm	36	−4.1 ± 6.1	39	−2.8 ± 4.1	−1.4	(-3.8, +0.9)	0.240	−1.1	(-3.2, +0.9)	0.282
Waist-hip ratio	36	−0.02 ± 0.07	39	−0.00 ± 0.05	−0.02	(-0.05, +0.00)	0.083	−0.02	(-0.05, +0.00)	0.083
Women										
3-months										
Weight, kg	33	−2.5 ± 3.1	26	−0.7 ± 2.1	−1.9	(-3.2, -0.6)	0.007	−1.6	(-2.8, -0.3)	0.016
BMI, kg/m^2^	33	−1.0 ± 1.2	26	−0.3 ± 0.8	−0.7	(-1.2, -0.2)	0.012	−0.6	(-1.1, -0.1)	0.020
Waist, cm	33	−3.5 ± 3.6	25	−1.9 ± 2.9	−1.6	(-3.4, +0.1)	0.074	−1.4	(-3.0, +0.2)	0.083
Waist-hip ratio	33	−0.01 ± 0.05	25	0.00 ± 0.03	−0.01	(-0.03, +0.01)	0.350	−0.01	(-0.03, +0.01)	0.295
6-months										
Weight, kg	33	−2.9 ± 4.5	25	−0.3 ± 3.4	−2.9	(-4.8, -1.0)	0.004	−2.3	(-4.0, -0.5)	0.013
BMI, kg/m^2^	33	−1.1 ± 1.7	25	−0.2 ± 1.4	−1.0	(-1.8, -0.2)	0.014	−0.8	(-1.5, -0.1)	0.025
Waist, cm	33	−4.0 ± 6.2	25	−2.0 ± 4.1	−2.0	(-4.8, +0.8)	0.172	−1.7	(-4.2, +0.8)	0.199
Waist-hip ratio	33	−0.02 ± 0.07	25	0.00 ± 0.05	−0.01	(-0.04, +0.02)	0.428	−0.01	(-0.04, +0.01)	0.371
Men										
3-months										
Weight, kg	5	−0.0 ± 2.4	14	−1.4 ± 1.8	+1.4	(-0.6, +3.3)	0.190	+1.3	(-0.3, +2.8)	0.135
BMI, kg/m^2^	5	−0.0 ± .08	14	−0.4 ± 0.6	+0.4	(-0.2, +0.9)	0.206	+0.4	(-0.1, +0.9)	0.102
Waist, cm	5	−1.5 ± 3.2	14	−2.0 ± 2.1	+0.3	(-2.0, +2.7)	0.785	+0.9	(-1.6, +3.4)	0.501
Waist-hip ratio	5	−0.04 ± 0.05	14	−0.00 ± 0.04	−0.04	(-0.07, -0.00)	0.037	−0.04	(-0.07, -0.01)	0.017
6-months										
Weight, kg	4	−0.2 ± 3.5	14	−2.4 ± 4.0	+2.4	(-1.3, +6.1)	0.227	+2.3	(-0.5, +5.1)	0.122
BMI, kg/m^2^	4	−0.1 ± 1.1	14	−0.7 ± 1.1	+0.6	(-0.5, +1.7)	0.281	+0.7	(-0.1, +1.5)	0.098
Waist, cm	3	−6.2 ± 3.6	14	−4.0 ± 4.0	−2.4	(-6.6, +1.8)	0.286	−0.2	(-3.8, +3.4)	0.902
Waist-hip ratio	3	−0.03 ± 0.06	14	0.00 ± 0.06	−0.03	(-0.09, +0.03)	0.338	−0.04	(-0.08, +0.00)	0.077

*Treatment effects were estimated using analysis of covariance (ANCOVA). For these analyses, the follow-up measurement was the dependent variable, treatment group was the independent variable and the baseline value of the measurement was included as the covariate

Because the distribution of males and females was found to differ significantly between treatment groups, post-hoc analyses with last-value carried forward were performed with sex included as a covariate. For the endpoints of weight and BMI, significant sex-by-treatment interaction effects were detected at both 3 months (*P* = .027 and P = .049) and 6 months (*P* = .020 and *P* = .044). Given these significant sex-by-treatment interaction effects, additional analyses were performed to assess all endpoints separately for males and females. For females, the telecoaching plus portion control intervention was associated with significant reductions in weight and BMI at both 3 (estimated treatment effect -1.6 kg, *P* = .016 and-0.6 kg/m^2^, *P* = .020) and 6 months (estimated treatment effect -2.3 kg, *P* = .013 and -0.8 kg/m^2^, *P* = .025). For males, the telecoaching plus portion control intervention was associated with a significant reduction in waist to hip ratio at 3-months (estimated treatment effect -0.04, *P* = .017).

### Changes in activity, diet and theory-based measures

We observed no significant overall difference in physical activity, dietary quality, or theory-based measures between the intervention and usual care groups. Among women, significant differences were observed for the change in METs per week based on the IPAQ (median change from baseline +778 versus -257 for telecoaching plus portion control versus usual care, respectively; *P* = .011), change in WEL total score (+12.5 versus -1.5, *P* = .041) and change in NEIS restructuring plans (+1.0 versus -1.0, *P* = .012) (Table 3).

**Table 3:** Secondary outcomes change from baseline to 3 months, overall, and according to sex

	Telecoaching + portion control		Usual care		
	N	median (25th, 75th)	N	median (25th, 27th)	*P* value*
All Subjects					
FFQ, total calories	38	−796, (-1303, -196)	41	−694 (-1371, -303)	0.662
IPAQ, total METs/week	39	+757 (-720, +2104)	40	+61 (-1071, +790)	0.090
WEL, total score	39	+12.0 (-4.0, +21.0)	40	+1.0 (-18.5, +16.25)	0.070
NEIS: Goal setting	39	+1.0 (-1.0, +3.0)	40	−1.0 (-2.0, +1.0)	0.027
NEIS: Restructuring plans	37	+1.0 (0.0, +1.0)	40	−1.0 (-2.0, +0.75)	0.006
NEIS: Relapse prevention	39	+2.0 (0.0, +3.0)	39	0.0 (-1.0, +3.0)	0.066
Women					
FFQ, total calories	33	−745 (-1233, -177)	26	−471 (-1224, -241)	0.598
IPAQ, total METs/week	34	+778 (-479, +2158)	26	−257 (-2690, +565)	0.011
WEL, total score	34	+12.5 (-4.0, +21.0)	26	−1.5 (-17.5, +14.0)	0.041
NEIS: Goal setting	34	+1.0 (-0.25, +3.0)	26	0.0 (-2.0, +0.25)	0.104
NEIS: Restructuring plans	32	+1.0 (0.0, +1.0)	26	−1.0 (-2.0, +0.25)	0.012
NEIS: Relapse prevention	34	+2.0 (+0.75, +3.75)	25	0.0 (-1.5, +3.5)	0.125
Men					
FFQ, total calories	5	−1316 (-2512, -607)	15	−707 (-1864, -342)	0.485
IPAQ, total METs/week	5	−5280 (-8611, +3598)	14	+480 (-248, +1248)	0.431
WEL, total score	5	+2.0 (-15.0, +31.0)	14	+6.5 (-23.0, +22.0)	0.963
NEIS: Goal setting	5	+1.0 (-3.5, +2.5)	14	−1.5 (-2.0, +1.0)	0.811
NEIS: Restructuring plans	5	+1.0 (−2.5, +15.0)	14	0.0 (−1.0, +1.0)	0.317
NEIS: Relapse prevention	5	−2.0 (−2.5, +1.5)	14	0.0 (−1.25, +1.25)	0.543

*FFQ* Food Frequency Questionnaire, *IPAQ* International Physical Activity Questionnaire, *NEIS* Neis Behavior Change Scale, *WEL* Weight Efficacy Life-Style Questionnaire

*Wilcoxon rank sum test

## Discussion

We observed that use of telecoaching with a portion control plate among obese primary care patients can produce positive change in body habitus and that changes depended upon sex. For women, the intervention was associated with weight loss maintained at 6 months as well as corresponding positive behavioral changes as assessed by the secondary outcome measures.

We observed an improvement in the waist to hip ratio at 3 months among the men in our study. Among men, waist to hip ratio may actually be a better predictor of mortality risk than weight loss [37, 38]. Interventions such as ours that have a clinically significant beneficial impact on waist to hip ratio may therefore potentially have a clinically significant impact on mortality risk in men.

A number of studies have recently highlighted possible differences between males and females in weight loss trials. Historically, men have been underrepresented in weight loss trials and many trials have enrolled females exclusively [39]. In the past, this has made it difficult to compare accurately the magnitude of effect in men versus women. A recent systematic review comparing weight loss interventions through diet and exercise found that men are generally more successful than women in weight-loss trials, but found no evidence that men and women should adopt different weight loss strategies [40].

To our knowledge, specific sex-based determinants influencing response to lifestyle intervention by motivational interviewing and setting behavior change goals have not been well characterized. However, our findings imply that telephone-based, motivational interview-based interventions in a primary care practice may be more successful among women.

The positive change in the NEIS and the WEL scores among the female participants who received telecoaching plus a portion control plate could indicate that these participants experienced improvements in eating self-efficacy as well as improvements in some constructs of behavioral change. Specifically, the significantly increased scores of the NEIS behavior change construct of restructuring indicates that these participants would be more comfortable restructuring their weight loss plan if success is not immediately achieved. Setting weight loss goals can have both positive as well as negative effects, and an increase in the construct of restructuring in behavioral change indicates a necessary skill in overcoming setbacks in behavioral change. Although the telecoaching intervention was not primarily focused on eating self-efficacy or restructuring, these appeared to be positive changes related to the overall effects of the intervention.

The strengths of this study include a randomized design and assessment of an intervention that is feasible in a primary care clinic. Telecoaching allows the behavioral interventions and frequent patient contact necessary to guide weight loss, and may utilize resources more efficiently than frequent clinic visits. The collaborative care model engages nonphysician members of the medical team. Minimal exclusion criteria of participants show that the intervention is successful primarily for obese women representative of those patients routinely seen in primary care clinics in regards to age, comorbidities, and baseline nutrition and physical activity status; however, generalizability is limited in regards to ethnicity, as a majority of participants was Caucasian.

Our study has limitations. First, we were limited by a relatively small sample size, low enrollment of males, and a random imbalance of males. We anticipated randomization would result in an even balance and did not stratify in anticipation of an imbalance. In addition, our study is limited by underrepresentation of minority populations who have higher rates of obesity [7].

Differences in patient contact time of the intervention existed between the two groups. Differences in contact time may potentially confound the assessment of efficacy of the telecoaching plus portion control plate intervention compared to a control intervention if contact time influenced outcome. Our study was not designed to assess the influence of contact time on our outcomes. Furthermore, we did not record the counseling sessions, nor did we require the counselors to follow a predefined script. This may hinder reproducibility but may increase generalizability.

Available evidence suggests that average weight losses of 2.5 kg to 5.5 kg at ≥2 years achieved with lifestyle intervention reduces the risk of diabetes by 30 % to 60 % [41]. Among women, we observed a significant weight loss of 2.9 kg in the complete case analysis at 6 months. We did not examine weight maintenance beyond this interval. Measures of physical activity and exercise intensity included the Seven Day Physical Activity Recall and the IPAQ. Although we did observe a significant difference in METS/week in the intervention group, these self-reporting tools are not the most reliable measures of physical activity. Finally, while the portion control plate was used as a tool to facilitate discussion with the telecoach, the study was not designed to isolate the relative impact of the plate versus the telecoaching intervention components.

## Conclusion

The recent AHA/ACC Obesity guidelines support the use of electronically delivered, including telephone, primary care weight loss programs focused on lifestyle interventions facilitated by a trained interventionist. This study supports telecoaching plus a portion control plate as a promising weight management strategy for obese women in primary care. Future research should evaluate whether this weight loss is maintained over time in these programs

## Additional files

Additional file 1:
**CONSORT Checklist.** (DOC 217 kb) Additional file 2:
**TIDieR Checklist.** (DOC 62 kb) Additional file 3:
**CONSORT Diagram.** (JPEG 187 kb)

## Abbreviations

BMI: body mass index
IPAQ: The International Physical Activity Questionnaire
METs: metabolic equivalents
FFQ: Food Frequency Questionnaire
WEL: Weight Efficacy Life-Style Questionnaire
WMSI: Weight Management Support Inventory
NEIS: Neis Behavior Change Scale.
