# Supplementary figures and images for: Telecoaching plus a portion control plate for weight care management: a randomized trial
Source: Trials. 2015 Jul 30;16:323. doi: 10.1186/s13063-015-0880-1 (PMC4518583; doi:10.1186/s13063-015-0880-1)

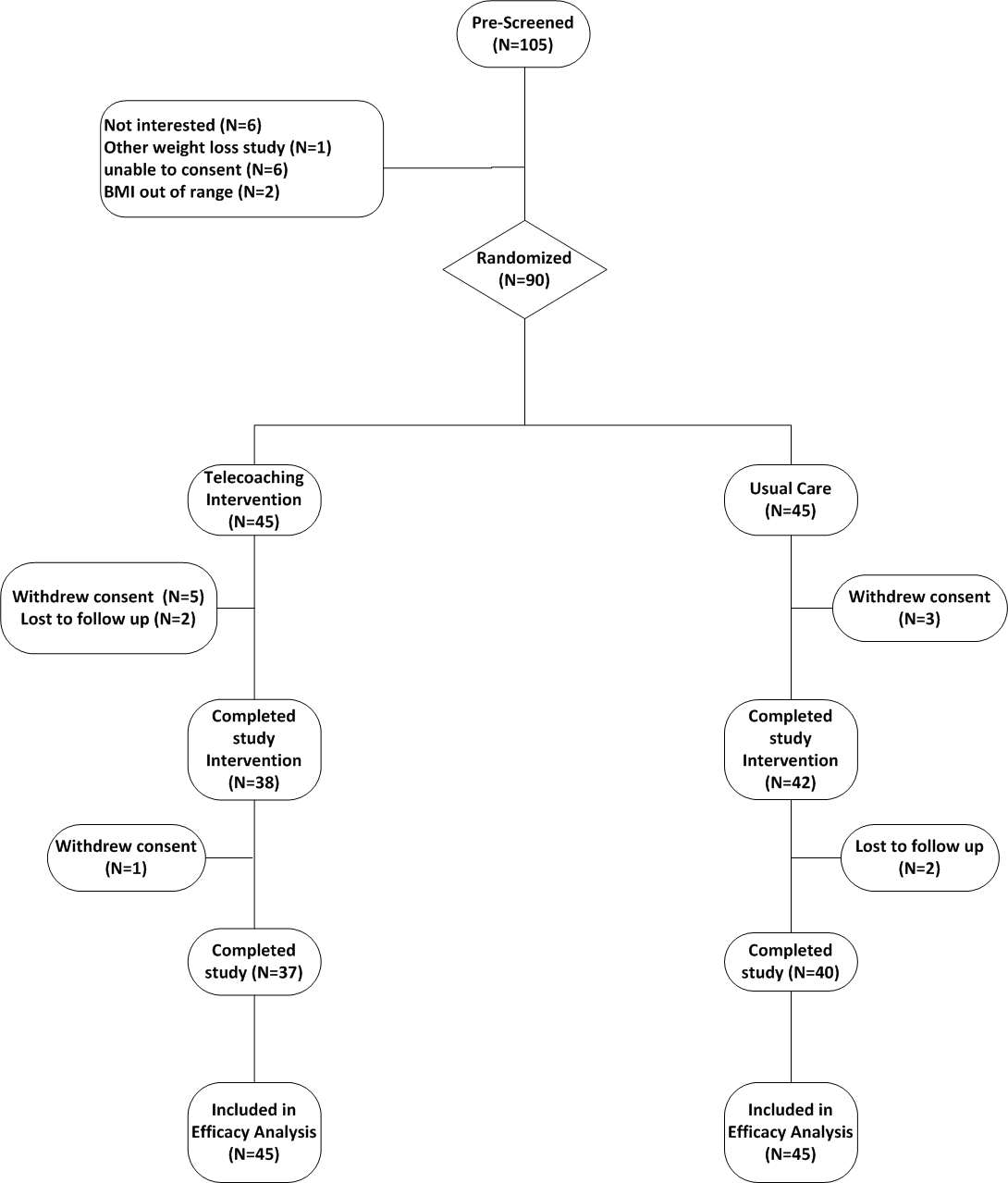

Supplement: Additional file 3: — CONSORT Diagram. (JPEG 187 kb) [file 13063_2015_880_MOESM3_ESM.jpeg]
